# Efficacy and Safety of Qinpi Tongfeng Formula Combined with Bloodletting Therapy in the Treatment of Acute Gouty Arthritis: A Study Protocol for a Randomized Controlled Trial

**DOI:** 10.1155/2022/3147319

**Published:** 2022-01-21

**Authors:** Hang Lu, Wei Liu, Yihua Fan, Wenliang Lv, Danna Yang, Chunliu Liu, Fangfang Lin

**Affiliations:** ^1^First Teaching Hospital of Tianjin University of Traditional Chinese Medicine, Tianjin 300193, China; ^2^National Clinical Research Center for Chinese Medicine Acupuncture and Moxibustion, Tianjin 300381, China; ^3^Guanganmen Hospital, China Academy of Chinese Medical Sciences, Beijing 100053, China; ^4^Gansu University of Chinese Medicine, Lanzhou 730050, Gansu, China

## Abstract

**Background:**

Acute gouty arthritis (AGA) is a common arthritis disease, with the characteristics of acute onset, severe condition, and poor prognosis. The conventional treatments have shown certain curative effects but are accompanied with many adverse reactions. The combination of orally taken Qinpi Tongfeng Formula (QPTFF) and bloodletting therapy could effectively alleviate arthralgia and joint swelling in AGA patients. However, there is a lack of high-quality randomized controlled trials (RCTs) to evaluate the clinical efficacy and safety of the combined therapy against AGA.

**Methods:**

This is a prospective, randomized, parallel controlled trial conducted in the First Teaching Hospital of Tianjin University of Traditional Chinese Medicine to explore the efficacy and safety of QPTFF combined with bloodletting therapy in the treatment of AGA. Eighty-six AGA patients meeting the inclusion and exclusion criteria will be randomly divided into the treatment group and control group in a 1 : 1 ratio using a randomization table. The investigators and the patients will not be blinded, while the outcome assessors and statisticians will be blinded to the allocation. Patients in the treatment group will take QPTFF and bloodletting therapy simultaneously, while patients in the control group will be instructed to orally take colchicine tablets. The primary outcome is the total effective rate, and the secondary outcomes are the pain changes after the first treatment, pain scores, complete pain relief time, joint symptom scores, TCM syndrome score, and laboratory test. SPSS22.0 will be used for statistical analysis. *Discussion*. This study will evaluate the clinical efficacy and safety of QPTFF combined with bloodletting therapy in the treatment of AGA, and the results of this study will provide reliable clinical evidence for the clinical use of QPTFF combined with bloodletting in the treatment of AGA. The trial is registered with ChiCTR2100048836.

## 1. Introduction

Gout, a crystal-induced arthritis, is caused by monosodium urate deposition because of purine metabolic disorder and/or reduced uric acid excretion [1]. Due to the disorder of purine metabolism or renal excretion, serum uric acid level increases abnormally, and finally, urate crystal deposition and acute persistent inflammatory reaction appear in the joints [2, 3]. Gout can be caused by both genetic factors and environmental factors. With the improvement of people's living standards and the change of diet structure and habits, the prevalence of gout increases largely, and the onset age of gout becomes younger and younger [4, 5]. According to the statistics of the World Health Organization (WHO), about 3.9% of people in the world are suffering from gout [6], with the ratio of male to female as 3 : 1 to 4 : 1 [7]. Gout patients are often accompanied by obesity, hypertension, nephropathy, hyperlipidemia, and other diseases, which have a serious burden on the health and economy of patients [8].

Acute gout arthritis (AGA) is the acute manifestation of gout, characterized by sudden and severe arthralgia with heat, redness, swelling, and restricted movement. At present, nonsteroidal anti-inflammatory drugs (NSAIDs) and colchicine are recommended as the first-line clinical treatments of AGA [9]. These medicines usually have good short-term efficacy, but long-term use may lead to gastrointestinal reactions, rashes, and even liver and kidney function injury [9, 10]. Hence, a multimodality therapy with fewer side effects to improve arthralgia and swelling of AGA is urgently needed, especially for some patients allergic to NSAIDs and colchicine.

Gout belongs to the paralysis syndrome (bi zheng) in traditional Chinese medicine (TCM), which is caused by dampness-heat accumulation and qi-blood stasis in the channels [11]. In China, the TCM treatment of gout has a long history. It was first discussed separately in Ge Zhi Yu Lun as early as 1347 AD. It has been reported that TCM formula combined with external treatment can alleviate the symptoms of gout quickly and delay the development of gout [12]. Qinpi Tongfeng Formula (QPTFF) is a derived compound prescription from Sheng Ji Zong Lu in 1117 AD according to the modern clinical application. The formula is composed of Cortex Fraxini (Qin Pi), Rhizoma Coptidis (Huang Lian), Radix Saposhnikoviae (Fang Feng), Semen Plantaginis (Che Qian Zi), Rhizoma Smilacis Glabrae (Tu Fu Ling), Rhizome Dioscoreae Hypoglaucae (Bi Xie), Radix Clematidis (Wei Ling Xian), and Herba Siegesbeckiae (Xi Xian Cao). QPTFF has shown the function of clearing dampness-heat, reducing swelling, and relieving pain [13]. A previous clinical study indicated that QPTFF had the same effect on alleviating arthralgia and joint swelling in AGA patients with an analgesic. Moreover, during the clinical trial, serious adverse reactions such as liver and kidney function damage and allergic dermatitis were not found [14]. Bloodletting therapy, one of the traditional external treatment methods, is the withdrawal of blood from a patient to prevent or cure illness and disease. During the process of bloodletting therapy, the operator uses a needle to prick into the skin at specific acupoints and suck a little blood through cupping to dredge the meridians, promote blood circulation, and relieve pain [15]. Clinical studies have found that bloodletting therapy can improve the local microcirculation and promote inflammatory absorption to relieve arthralgia [16]. However, there is still a lack of high-quality clinical study to verify the clinical efficacy of the combination of the two therapies in the treatment of AGA. Hence, in this study, we try to conduct a randomized, controlled trial to analyze the efficacy and safety of QPTFF combined with bloodletting therapy in the treatment of AGA.

## 2. Materials and Methods

### 2.1. Study Design

**Figure 1:**
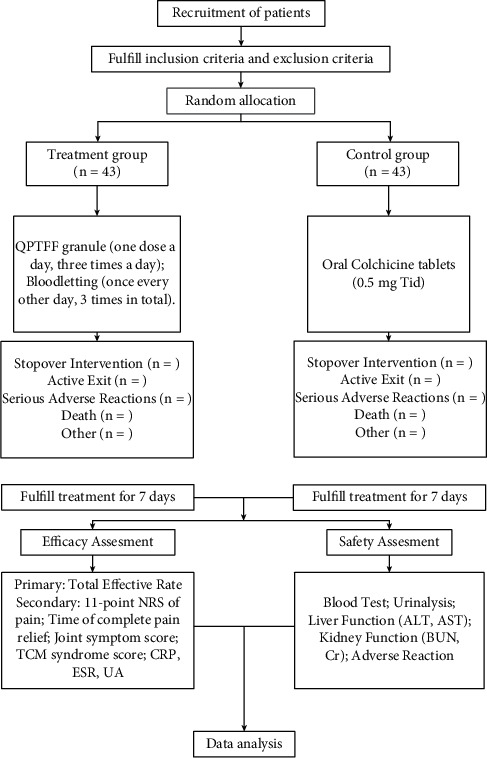
The flow chart of this study.

**Figure 2:**
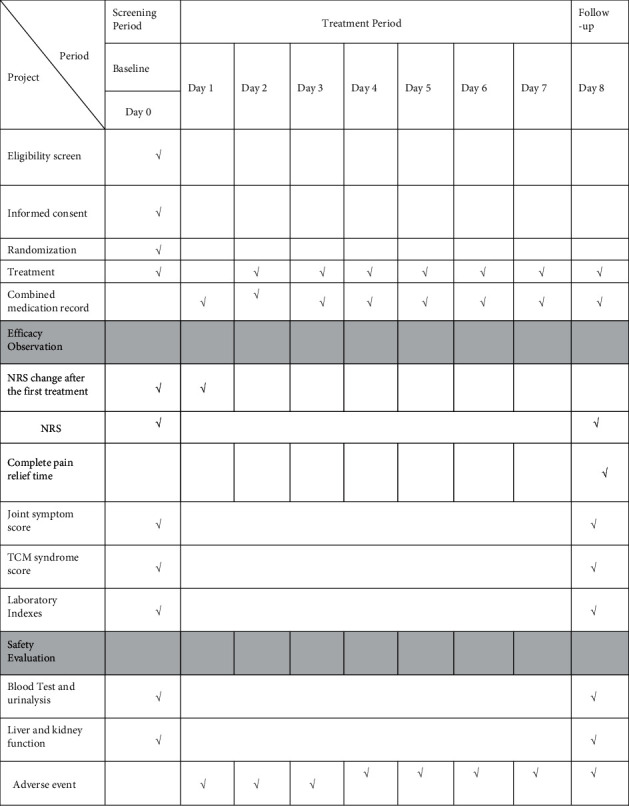
Patient follow-up process.

This trial is designed as an open-label, prospective, randomized, controlled, and parallel-group study. It will be conducted in the First Teaching Hospital of Tianjin University of Traditional Chinese Medicine from August 1, 2021 to October 30, 2021.  Figure 1 shows the research flow chart, and the details of patient follow-up are summarized in Figure 2. The research follows the latest Consolidated Standards of Reporting Trials (CONSORT 2017) and the Standard Protocol Items: Recommendations for Interventional Trials (SPIRIT) 2013 statement (the SPIRIT checklist is provided in Table S1).

### 2.2. Ethics and Registration

This study protocol will be conducted in accordance with the *Declaration of Helsinki* and the *Ethical Guidelines for Clinical Research*. This study has been approved by the ethics committee of the First Teaching Hospital of Tianjin University of Traditional Chinese Medicine (Ethics number: TYLL2021 [Z] 014), and it has been registered in the Chinese Clinical Trial Registry (Registration number: ChiCTR2100048836).

### 2.3. Participants

Participants will be recruited from outpatients in the rheumatology department of the First Teaching Hospital in Tianjin University of Traditional Chinese Medicine via posters and WeChat. Recruitment members in the rheumatology department will be in charge of the recruitment and registration of the participants meeting the inclusion and exclusion criteria. Written informed consent will be obtained from participants or their legal representatives, and the personal information will be kept with utmost secrecy.

### 2.4. Diagnostic Basis

The diagnosis of AGA is according to the diagnostic criteria of the American College of Rheumatology in 2015 [17]. The syndrome diagnosis of dampness-heat accumulation syndrome in TCM is according to the guidelines for the combined diagnosis and treatment of gout and hyperuricemia issued by the Chinese Society of TCM in 2020 [18], with two primary symptoms or one primary symptom plus two secondary symptoms in combination with the tongue and pulse. The primary symptoms include redness, swelling, heat, and pain in the joint and sudden onset of arthralgia, and the secondary symptoms include poor joint movement, fever, and dysphoria. The tongue should be red, with yellow, greasy or thick coating, and the pulse should be slippery.

### 2.5. Recruitment Criteria

The inclusion criteria are as follows:

1. Meeting the diagnostic criteria of AGA of the American Society of Rheumatology in 2015
2. Meeting the TCM diagnostic criteria of gout with dampness-heat accumulation syndrome
3. 72 hours within the onset of AGA
4. With moderate, severe, or extreme arthralgia and the score of the numerical rating scale (NRS) ≥4
5. Without taking any other medicines for AGA 72 hours before the enrollment
6. Without taking any uric acid-lowering medicines during the last 2 weeks
7. Age of 18–70 years
8. With signed informed consent form

### 2.6. Exclusion Criteria

The exclusion criteria are as follows:

1. Secondary gouty arthritis caused by other factors (e.g., renal failure, chemotherapy or radiotherapy, and drugs)
2. With rheumatoid arthritis, psoriatic arthritis, ankylosing spondylitis, knee osteoarthritis, and other arthritis
3. With multiple joints involved (>4 joints)
4. Alanine aminotransferase (ALT), alanine aminotransferase (AST), or creatinine (Cr) 1.5 times higher than the upper limit of normal [19]
5. History of allergy to any ingredients of the medicines in both groups
6. Women in pregnancy or lactation
7. With peptic ulcer and bleeding
8. Having participated in other clinical trials in the past month
9. With serious mental illness, unable to express accurately or take medicine on time, and unable to cooperate

### 2.7. Termination Criteria

1. Participants experiencing adverse events (e.g., cardiovascular embolism, gastrointestinal reaction, and severe liver and kidney dysfunction) or other complications that the investigators consider it necessary to terminate trial treatment
2. Participants could not benefit or even get worse from the trial treatment that the investigators deem necessary to terminate trial treatment
3. Participants stop or discontinue the medication or take other drugs at will without authorization
4. Participants are unwilling or impossible to continue the trial and request to withdraw and terminate the trial

### 2.8. Sample Size

PASS15.0 is used to calculate the sample size. The study is suitable for the noninferiority test. According to our preliminary study, the total effective rate of QPTFF combined with bloodletting is 95%, and the total effective rate of colchicine is 90% [20]. Taking *α* = 0.05, *β* = 0.2, a ratio of 1 : 1, and the boundary value = −0.1, the sample size of the two groups is 78 cases. Considering a loss to follow-up of 10%, 86 cases are finally needed, with 43 cases in each group.

### 2.9. Randomization and Allocation Concealment

Participants will be randomly divided into the treatment group and control group in a 1 : 1 ratio using a randomization table. The randomization sequence will be generated by an independent statistician with Excel 2013 software. The random numbers will be placed in opaque, sealed envelopes and kept in a safe place until the study is completed. Chunliu Liu will be responsible for the enrollment and assignment of the participants.

### 2.10. Blinding

Due to the limitation of the intervention program, the blinding of bloodletting therapy is quite difficult to achieve. The investigators and the patients will not be blinded, while the outcome assessors and statisticians will be blinded to the allocation.

### 2.11. Intervention

In the treatment group, patients will take QPTFF and bloodletting therapy simultaneously. The QPTFF will be provided as granules, composed of Cortex Fraxini (Qin Pi) 30g, Rhizoma Coptidis (Huang Lian) 10g, Semen Plantaginis (Che Qian Zi) 20g, Rhizoma Smilacis Glabrae (Tu Fu Ling) 80g, Radix Clematidis (Wei Ling Xian) 30g, Herba Siegesbeckiae (Xi Xian Cao) 30g, and Radix Saposhnikoviae (Fang Feng) 10g, prepared by Sichuan New Green Pharmaceutical Technology Development Co., Ltd. The patients will be instructed to orally take the granules one dose a day by dissolving them in 50 mL warm water, three times a day, for 1 week. The bloodletting therapy will be performed by qualified members who have more than 3-year experience in bloodletting therapy after being retrained for standard management for the study. The main acupoints are Ashi points (affected side), SP10 (Xuehai) (bilateral), SP9 (Yinlingquan) (bilateral), ST36 (Zusanli) (bilateral0), BL40 (Weizhong) (bilateral), BL20 (Pishu) (bilateral), and SP6 (Sanyinjiao) (bilateral). At the same time, local points will be selected. For the first metatarsophalangeal joint, SP3 and LR3 will be added; for medial malleolus, KI7 will be added; for the lateral ankle, BL57 will be added. During the bloodletting operation, the patient will take the prone position. The operator disinfects the acupoints with 75% alcohol, pricks the acupoints with the sterilized lancet, place a fire cup on the point for 5 min to suck the blood, and then disinfects the skin with 75% alcohol. The bloodletting therapy will be performed once every other day, 3 times in total.

In the control group, patients will be instructed to orally take colchicine tablets (Guangdong BIDI Pharmaceutical Co., Ltd., China, H20113208), 0.5 mg once, 3 times a day, for 1 week.

If the pain in the two groups is severe and unbearable during the treatment, etocoxib (Hangzhou MSD Pharmaceutical Co., Ltd., China, J20180059) 120 mg, once a day, will be used. The patients will be asked to record the dosage and administration of the medicine.

### 2.12. Outcome Measures

The primary outcome is the total effective rate according to the *Guiding Principles for Clinical Research of New Traditional Chinese Medicine*, as divided into clinical cured, remarkably effective, effective, and ineffective [21, 22]. Clinical cured means that the clinical symptoms and signs disappear or basically disappear, and the curative effect index decreases more than 95% (included). Remarkably effective means that the clinical symptoms and signs are significantly improved, and the curative effect index is reduced more than 60% (included) but less than 95%. Effective means improvement of clinical symptoms and signs, and a reduction of curative effect index of more than 30% (included) but less than 60%. Ineffective means that the clinical symptoms and signs are not improved or even aggravated, and the curative effect index is reduced less than 30%. Total effective rate = (clinical cured number + remarkably effective number + effective number)/total × 100%. The total effective rate of the treatment of patients will be evaluated after the treatment by Fangfang Lin.

The secondary outcomes are the pain changes after the first treatment, pain scores, complete pain relief time, joint symptom scores, TCM syndrome score, and laboratory test, as measured by Wenliang Lv and Danna Yang.

1. The pain changes after the first treatment will be evaluated by the 11-point Numerical Rating Scale (NRS) [23]. The scale includes 0–10 points to represent the different degrees of pain, and the grading standards of pain degree are 0 for painless, 1–3 for mild pain, 4–6 for moderate pain, and 7–10 for severe pain. In the treatment group, the patients will be instructed to take QPTFF once at the first visit and then to accept bloodletting therapy. After the bloodletting therapy, the changes of NRS scores at 0.5 h, 1 h, 1.5 h, and 2 h will be recorded to evaluate the immediate efficacy of TCM therapy. In the control group, after taking colchicine for the first time, the NRS scores of patients will be recorded at 0.5 h, 1h, 1.5 h, and 2h.
2. Pain scores will be measured by the NRS on day 0 and day 8 (follow-up).
3. Complete pain relief time is defined as the number of days required from the initial treatment to the complete pain relief measured by the NRS at the end of the study.
4. Joint symptom score is used to evaluate the tenderness, redness, swelling, and mobility of joints. The higher the score, the heavier the symptom is. The scores will be recorded on day 0 and day 8 (follow-up).
5. TCM syndrome score, according to the *Guiding Principles for Clinical Research of New Traditional Chinese Medicine* [22], includes TCM syndromes such as joint swelling, skin temperature, thirst, and yellow urine. The higher the score, the more serious the condition is. The scores will be recorded on day 0 and day 8 (follow-up).
6. Laboratory tests include C-reactive protein (CRP), erythrocyte sedimentation rate (ESR), and uric acid (UA). The results will be recorded on day 0 and day 8 (follow-up).

### 2.13. Safety Evaluation

Blood test, urinalysis, liver function, and renal function (urea nitrogen and serum creatinine) will be measured on day 0 and day 8 to evaluate the safety of treatment. At the same time, patients are required to record any adverse reactions during the study and report to the investigator at any time. Details of all adverse events will be recorded in case report dorms (CRFs), including time, degree and duration, suspected causes, measures, and results. After the treatment, the adverse reactions of the two groups will be counted.

### 2.14. Data Management and Quality Control

Any modification or change of the protocol will be approved through the formal procedures of the ethics committee of the First Affiliated Hospital of Tianjin University of Traditional Chinese Medicine. Independent clinical research assistants will regularly review the study progress. CRFs will be used in data collection to record demographics, assessment, and reasons for drop-out. All CRFs will be stored in an independent storage room to protect confidentiality. Without the written permission of the supervisor, the participant's information will not be disclosed and shared. At the end of the study, the investigator will submit the CRFs to the data management committee, and the investigators cannot modify the data.

### 2.15. Statistical Analysis

Efficacy evaluation will be determined by full analysis set (FAS) and per-protocol set (PPS), and safety evaluation will be based on safety set (SS). The statistical evaluation of FAS will follow the intent-to-treat (ITT) principle. The last observation carried forward (LOCF) method will be used to estimate the missing values of main variables. The collected data will be statistically analyzed by using the SPSS22.0 software (International Business Machines Corporation, New York, USA). For continuous data, they will be represented as mean ± standard deviation and frequency or percentage for categorical data. For the primary outcome, the chi-square test will be used. For secondary outcomes, independent samples *T* test or Mann–Whitney *U* test for intergroup comparison will be used for pain scores, complete pain relief time, joint symptom scores, TCM syndrome score, and laboratory test and generalized linear models or repeated measures analysis of variance for the pain changes after the first treatment after considering the normality and homogeneity. Statistical testing is two-sided, and *P* < 0.05 is considered statistically significant.

## 3. Discussion

The main TCM syndrome of AGA is dampness-heat accumulation syndrome [24], which is characterized by arthralgia, swelling, redness, and fever, accompanied by dysphoria, thirst, and yellow urine [21]. Based on the theory of TCM, the treatment of AGA with dampness-heat accumulation syndrome should follow the principle of clearing dampness-heat and dredging meridians. The mechanism is to inhibit inflammatory factors in the joint fluid, reduce serum uric acid, promote uric acid excretion, regulate immune function, and block peripheral nerve pain [12].

Traditional Chinese medical formula has the characteristics of multicomponent, multitarget, and multilevel in the treatment of AGA [24]. Modern pharmacological studies have found that aescin, the effective component of the main herb Cortex Fraxini (Qin Pi) in QPTFF, can inhibit the overexpression of inflammatory factors and catabolic genes, promote the upregulation of cartilage-specific genes and scavenge reactive oxygen species (ROS), to show anti-inflammatory, antioxidant, and protective effects on chondrocytes [26, 27]. Rhizoma Smilacis Glabrae (Tu Fu Ling) can inhibit uric acid production and promote uric acid excretion, reduce capillary permeability, improve microcirculation, and improve joint swelling [28]. Its effective component, resveratrol, can inhibit IL-1*β* secretion and downregulation of NF-*κ*B p65 expression and reduce the production of inflammatory factors and chemokines and inflammatory cell infiltration [29]. Herba Siegesbeckiae (Xi Xian Cao) has anti-inflammatory and analgesic effects. It can regulate joint inflammation and pain by controlling inflammatory factors in joints and reducing immune response [30]. Rhizome Dioscoreae Hypoglaucae (Bi Xie) can regulate the expression of inflammatory factors, reduce uric acid in the blood, and protect renal function [31]. In a word, a variety of effective components in QPTFF could play therapeutic roles against AGA.

In China, bloodletting therapy is often used to treat AGA with dampness-heat accumulation syndrome. Clinical studies have found that bloodletting therapy can upregulate the local anti-inflammatory factors IL-4 and IL-10, mediate TLR4/IL-1 signal pathway, regulate immune response, reduce inflammatory cell infiltration [15], and relieve pain. Stimulating BL40 can promote uric acid excretion and improve microcirculation [32], and stimulating SP6 and SP9 can inhibit the synthesis of inflammatory cytokines and reduce TNF-*α* in serum to play anti-inflammatory and detumescence roles [33]. ST36 could effectively function in reducing inflammation in many studies [34, 35], and SP6 can reduce the production and secretion of pain-causing factors and monoamine transmitters in AGA rats to relieve pain [33, 36].

The combination of QPTFF and bloodletting therapy has positive and safe effects on AGA patients, but there is still a lack of high-quality clinical study. The previous reported RCTs of TCM in the treatment of AGA showed many limitations. For example, the treatment in the control group was not a classic control such as NASID or colchicine [37]. In addition, those RCTs did not observe the immediate efficacy of TCM treatment [38]. In our study, we try to observe the pain changes within 2 hours right after the patients take the combined therapy, which is helpful to analyze the immediate effect of TCM therapy. Therefore, this study intends to explore the efficacy of QPTFF combined with bloodletting therapy through a high-quality RCT. We take the changes of patients' clinical symptoms and signs as the main outcome indicators, including joint symptoms and TCM syndromes. In addition, we use laboratory indexes to evaluate the efficacy of TCM therapy objectively and liver and kidney function, blood test, and urinalysis to evaluate the safety of the treatment.

Still, there are also some limitations in this study. Due to the characteristics of bloodletting therapy, the operator and the patients cannot be blinded, which may impact the research results. Due to the lifestyle and diet habits of the population in Tianjin, the similar baseline information of the body mass index (BMI), history of gout, and uric acid level in both groups may lead to a single sample and regionalization. Because we just observed the short-term curative effect on AGA, it was difficult to observe the long-term efficacy in two groups after the trial.

## Data Availability

After the study, the datasets used and analyzed in the current study are available from the corresponding author on reasonable request (Wei Liu: fengshiliuwei@163.com). Also, the data will be available in the “Chinese Clinical Trial Registry” (http://www.chictr.org.cn). The registration number is ChiCTR2100048836.

## Abbreviations

NSAIDs: Nonsteroidal anti-inflammatory drugs
CONSORT: Consolidated Standards of Reporting Trials
RCT: Randomized controlled trial
QPTFF: Qinpi Tongfeng Formula
ROS: Reactive oxygen species
ALT: Alanine aminotransferase
AST: Alanine aminotransferase
Cr: Creatinine
CRP: C-reactive protein
ESR: Erythrocyte sedimentation rate
UA: Uric acid
NRS: Numerical rating scale
CRF: Case report form
FAS: Full analysis set
PPS: Per-protocol set
SS: Safety set
ITT: Intent-to-treat
LOCF: Last observation carried forward.

## Supplementary Materials

Supplementary Materials Table S1: SPIRIT 2013 checklist: recommended items to address in a clinical trial protocol and related documents.
